# Ultrafast imaging of terahertz electric waveforms using quantum dots

**DOI:** 10.1038/s41377-021-00693-5

**Published:** 2022-01-01

**Authors:** Moritz B. Heindl, Nicholas Kirkwood, Tobias Lauster, Julia A. Lang, Markus Retsch, Paul Mulvaney, Georg Herink

**Affiliations:** 1 Experimental Physics VIII – Ultrafast Dynamics, University of Bayreuth, Bayreuth, Germany; 2 ARC Centre of Excellence in Exciton Science, School of Chemistry, University of Melbourne, Melbourne, Australia; 3 Physical Chemistry I, University of Bayreuth, Bayreuth, Germany

**Keywords:** Nanophotonics and plasmonics, Quantum dots

## Abstract

Microscopic electric fields govern the majority of elementary excitations in condensed matter and drive electronics at frequencies approaching the Terahertz (THz) regime. However, only few imaging schemes are able to resolve sub-wavelength fields in the THz range, such as scanning-probe techniques, electro-optic sampling, and ultrafast electron microscopy. Still, intrinsic constraints on sample geometry, acquisition speed and field strength limit their applicability. Here, we harness the quantum-confined Stark-effect to encode ultrafast electric near-fields into colloidal quantum dot luminescence. Our approach, termed Quantum-probe Field Microscopy (QFIM), combines far-field imaging of visible photons with phase-resolved sampling of electric waveforms. By capturing ultrafast movies, we spatio-temporally resolve a Terahertz resonance inside a bowtie antenna and unveil the propagation of a Terahertz waveguide excitation deeply in the sub-wavelength regime. The demonstrated QFIM approach is compatible with strong-field excitation and sub-micrometer resolution—introducing a direct route towards ultrafast field imaging of complex nanodevices in-operando.

## Abstract (web summary)

Novel Quantum-probe Field Microscopy (QFIM) captures ultrafast movies of Terahertz electric waveforms inside microstructures using fluorescent quantum dots.

## Graphical abstract

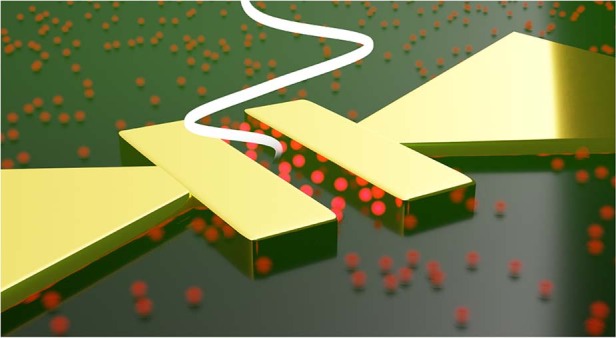

## Introduction

The detection of radiation—including human vision—is typically sensitive to the energy carried by an electromagnetic wave rather than its fields. Heinrich Hertz succeeded to prove the existence of electromagnetic fields by conversion into incoherent visible fluorescence^1^. Today, electric waveforms can coherently be sampled with ultrashort laser pulses^2–4^ to directly access the temporal signatures of charge motion and quasi-particle excitations in condensed matter systems up to the visible spectrum^5^. Yet, relevant field distributions are often confined to microscopic scales significantly below the diffraction limit—arising from inhomogeneity of materials, microstructures or intrinsic confinement of light-matter excitations^6–8^. Only a few approaches spatially resolve local electric near-field waveforms up to multi-Terahertz frequencies, including raster-scanned photoconductive switches and electro-optic microscopy^9–13^. Enhanced resolution is provided by scattering near-field optical microscopy^14–17^, THz-driven scanning tunneling microscopy^18,19^ and recently emerging ultrafast electron microscopy^20–22^. THz-induced visible luminescence has been employed for imaging spatial field distributions via temporally cumulated effects of strong local fields^23–26^. Sampling THz electric waveforms in the time-domain using visible fluorescence appears highly desirable as it bears numerous prospects including the access to nanoscopic scales, 3D geometries, high-speed acquisition, and compatibility with strong local fields inside active and nonlinear-driven devices^7,27–30^.

**Fig. 1:**
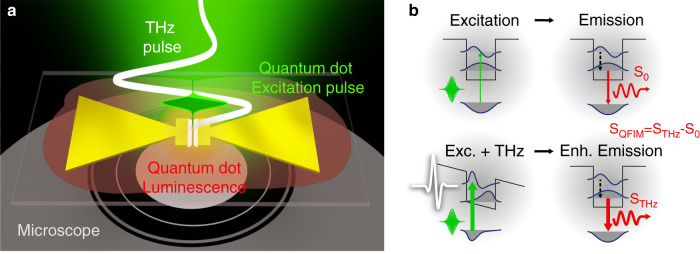
Quantum-Probe Field Microscopy (QFIM). **a** Imaging of THz electric near-fields in a fluorescence microscope using quantum dot (QD) luminescence. The absorption of ultrashort visible sampling pulses (green) is modulated via the quantum-confined Stark effect in a layer of nanocrystals (red). **b** The THz-induced change in the QD band structure can increase the absorption and translates to enhanced luminescence emission, accessible by optical microscopy. The modulated fluorescence yield *S*_QFIM_ *=* *S*_THz_*−S*_0_ encodes the instantaneous local electric field and snapshot images resolve the spatio-temporally evolution of the near-field waveform

Here, we demonstrate ultrafast far-field imaging of THz electric near-fields using fluorescence microscopy. We capture visible photons from local quantum dot probes and acquire stroboscopic movies of electric near-field evolutions. The scheme employs the quantum-confined Stark effect (QCSE)^31–33^, encoding electric near-fields into far-field luminescence modulations via variations of photo-absorption, illustrated in Fig. 1. THz-induced quasi-instantaneous interactions were previously reported for diverse 0D-quantum systems^26,34,35^. Harnessing this mechanism, we perform spatially resolved time-domain spectroscopy, and demonstrate the imaging capabilities by resolving the ultrafast electric waveforms of (a) the localized THz resonance of a bowtie antenna and (b) the propagating THz gap excitation inside a micro-slit waveguide. Akin to plasmonics in the visible and near-infrared spectrum, these highly localized excitations arise from collective oscillations of the electron plasma constrained by sub-wavelength geometries.

## Results

Our experiments are based on two-color excitation using single-cycle Terahertz pulses to drive phase-stable near-fields and visible fs-pulses to excite the quantum dot probes, see Fig. 1a. The incident THz pulses at electric field strengths up to 400 kV/cm are enhanced in lithographically patterned gold structures. Colloidal CdSe-CdS core-shell nanocrystals, similarly used in voltage sensing applications^36,37^, are deposited as a homogeneous layer of quantum-probes via drop-casting. Luminescence is excited via wide-field illumination in the image plane of a fluorescence microscope with ~150 fs pulses at wavelengths around 500 nm. We acquire differential images of the emission yield with a CCD camera in the presence and absence of THz excitation. The difference signal, which we refer to as the QFIM signal *S*_QFIM_ in the following, represents the crucial observable for instant local fields.

**Fig. 2:**
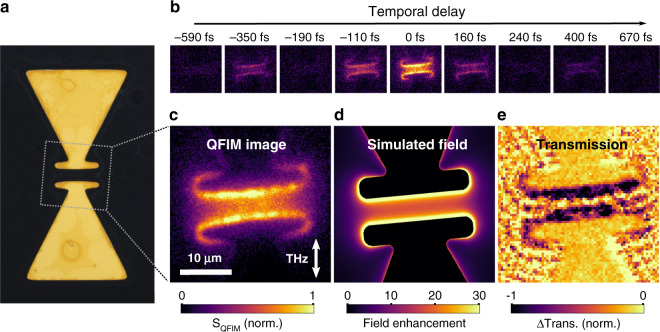
Evolution of THz near-fields in a resonant bowtie antenna. **a** Optical micrograph of the THz bowtie resonator. **b** A series of subsequent microscopic snapshots at selected delays tracks the THz near-field with sub-cycle temporal resolution (incident THz polarization as indicated). **c** QFIM snapshot at the peak local field at Δ*τ* **=** 0 fs. **d** The simulated spatial near-field distribution at resonance in the gap region closely resembles the QFIM signal in (**c**). **e** A snapshot acquired in transient transmission contrast (Δ*τ* = 0 fs) corroborates the field-driven absorption modulation as the origin of the QFIM signal

First, we follow the ultrafast near-field evolution inside a THz antenna structure, shown in Fig. 2a, with sub-cycle temporal resolution by acquiring a sequence of snapshot images at increasing delays between THz and visible pulses. Figure 2b shows nine exemplary frames out of a series with temporal separation of Δ*τ* = 30 fs (full movie in Media 1). We observe a strong enhancement in the antenna gap and close to the terminal bars (THz polarization ~0° to the antenna axis). The signal is maximized at the edge of each antenna leg and decays symmetrically towards the center of the bowtie as apparent in the snapshot at Δ*τ* = 0 fs in Fig. 2c, demonstrating a spatial resolution of ~2 µm (see Supplementary Information). This pattern visually matches finite-element simulations of the THz electric near-field, shown in Fig. 2d, and strongly depends on the incident polarization (data for THz polarization ~90° to the antenna axis in Supplementary Information). Based on the simulated field enhancement and the incident peak field of ~400 kV/cm, we estimate a maximum near-field strength of ~10 MV/cm.

**Fig. 3:**
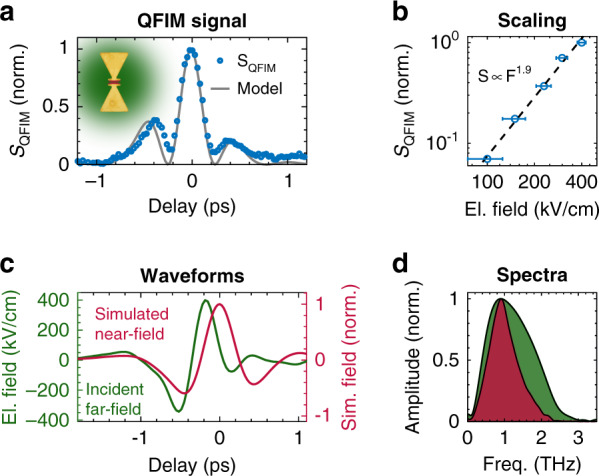
QFIM signal and near-field waveform inside a bowtie antenna. **a** Local QFIM signal in the gap of the bowtie (blue circles) and the modeled temporal luminescence evolution (gray) based on the incident waveform. **b** Scaling of the peak QFIM signal (circles) as a function of the maximum incident field strength. **c** Comparison of the driving far-field waveform (green) and the simulated local near-field inside the bowtie gap (red). **d** Corresponding spectra of both waveforms shown in (**c**)

Analyzing the QFIM signal inside the gap, we demonstrate the extraction of local electric waveforms and characterize the temporal response of the bowtie antenna. As a prerequisite, we study the relation between the maximum field strength *F* and the peak signal of *S*_QFIM_. Measurements with varying incident field strengths yield the dependence *S*_QFIM_ ∝ *F*^1.9^ for the quantum dots used in the experiment, as evident in the double-logarithmic representation in Fig. 3b. Thus, the peak signal scales nonlinearly with the maximum incoming field^34^. Employing the rectifying relation and the incident far-field waveform—obtained from calibrated conventional electro-optic sampling (EOS)—, we simulate the local near-field and the resulting QFIM signal using a finite-element time-domain simulation of the structure and find close agreement with the experimental QFIM trace, see Fig. 3a. The comparison of the incident THz waveform and the simulated near-field evolution is shown in Fig. 3c with corresponding spectra in Fig. 3d. Alternatively, a reconstruction of the near-field in a resonator can be obtained by adapting a single resonance model to the QFIM data, as shown in the Supplementary Information. Depending on the signal quality, direct extraction of near-field waveforms appears feasible via recovery of the polarity and reversal of the nonlinear QFIM signal.

The underlying mechanism enabling the QFIM scheme relies on THz-driven modulations of the electronic band structure in low-dimensional quantum systems^31,32^, i.e., the QCSE in semiconductor nanocrystals^33^. The altered electron and hole wavefunctions induce a quasi-instantaneous change of the optical transition dipole moment. As a result, the photoabsorption may be reduced or enhanced depending on the visible excitation frequency and the accessed electronic states, as previously resolved via transient absorption spectroscopy^35^. We spatially map these changes via luminescence emission microscopy. Specifically, we note that irrespective of much longer luminescence lifetimes (~10 ns), the temporal sampling resolution is exclusively governed by the ultrafast absorption process. This quasi-instantaneous absorption can alternatively be accessed via transient absorption imaging of the antenna, as shown, e.g., for Δ*τ* = 0 fs in Fig. 2e, yielding a pattern complementary to the QFIM signal.

**Fig. 4:**
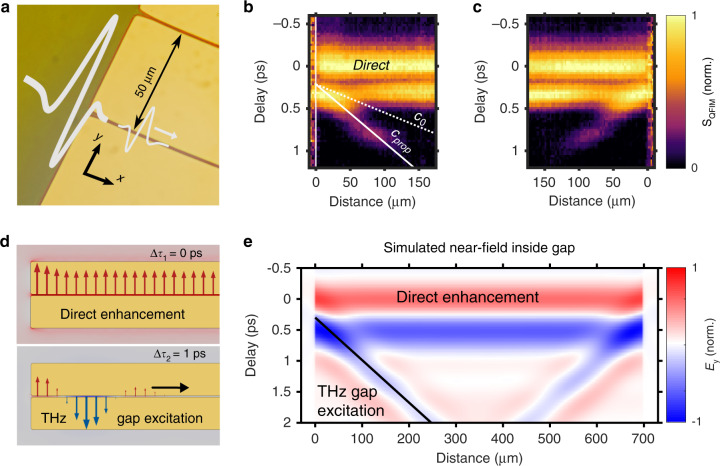
Temporal imaging of a propagating THz gap excitation. **a** Launching of a THz waveguide excitation in a micro-slit driven at normal incidence (2 µm gap). **b** 2D representation (*x*, Δ*τ*) of the normalized QFIM waveforms along the slit temporally disentangles the spatially homogeneous direct response of the slit (direct field enhancement) and a THz gap excitation propagating along the waveguide. The peak electric field inside the waveguide propagates with a velocity below *c*_0_ (dashed line) as quantitatively predicted by time-domain electromagnetic simulation (*c*_prop_, solid line). **c** The reversed propagation direction is observed via QFIM for excitation at the opposite side of the waveguide. **d** Simulated electric field distributions for two temporal delays illustrate the homogeneous direct enhancement (Δ*τ*_1 _= 0 ps) and the propagating THz excitation (Δ*τ*_2 _= 1 ps). **e** 2D representation (*x*, Δ*τ*) of the simulated electric field evolution along the gap in correspondence to (**b**, **c**)

Now, we demonstrate the field-resolved tracking of propagating ultrafast THz excitations using the QFIM scheme. Specifically, we spatio-temporally resolve a THz wavepacket traveling along the subwavelength slit of a gold waveguide, as depicted in Fig. 4a. We map the temporal evolution of the QFIM signal along the gap in a 2D representation (*x*, Δ*τ*) in Fig. 4b, resolving two distinct features: First, the horizontal lines arise from the direct field enhancement inside the gap extending over the THz focus. Subsequently, the tilted feature reveals the propagation of a THz gap excitation with a velocity *c*_prop_ below *c*_*0*_ emerging from the left edge of the structure. Such propagating plasmonic excitations are confined inside a subwavelength slit and provide the basis for ultrafast circuits—enabling the routing, nanofocusing, and enhancement of infrared radiation^12,38–42^. We corroborate our finding with a time-domain electromagnetic simulation of the ultrafast interaction (see “Materials and methods”), yielding the launching of a THz wavepacket from the edge with a propagation velocity *c*_prop_ (white solid line in Fig. 4b) in agreement with the experimental QFIM dataset. This gap excitation manifests as a spatially oscillating electric field distribution along the slit—in contrast to the unidirectional field of the direct enhancement, illustrated by the simulated fields at two exemplary temporal delays (Δ*τ*_1 _= 0 ps, Δ*τ*_2 _= 1 ps) in Fig. 4d. In correspondence to Fig. 4b, c, we present the simulated electric near-fields as a spatio-temporal map in Fig. 4e. The simulation yields a phase velocity of the waveguide excitation between the vacuum and the substrate of *c*_prop_ ~ *c*_0_/2. Moreover, we also reproduce the experimentally observed interference of the direct and the propagating pulses. We attribute the different propagation lengths of experiment and simulation to the idealized homogeneous microstructure assumed in the model^43^. Furthermore, the simulation yields a second gap excitation at the opposite side of the THz waveguide. We experimentally resolve this feature in a QFIM measurement acquired at the right side of the waveguide in Fig. 4c.

## Discussion

We introduce Quantum-probe Field Microscopy to image ultrafast electric near-field waveforms in the time-domain. Our approach utilizes the encoding of momentary THz-fields onto the visible emission of nanocrystals and far-field fluorescence imaging. The underlying THz field-driven and quasi-instantaneous QCSE provides a direct link between the luminescence observable and the local electric fields. On this basis, we demonstrate the time-resolved microscopy of near-field waveforms inside a single bowtie antenna—a building block of ultrahigh-frequency devices, metamaterials, and strong-field light-matter interaction experiments^27,28^. Moreover, we observe THz propagation inside a gap deeply in the sub-wavelength regime and, thus, introduce the ultrafast sampling of propagating electric fields inside confined structures in the time domain. These results motivate the application of QFIM for imaging electric waveforms of surface excitations, including THz phonon and plasmon polaritons on bulk surfaces and 2D heterostructures^44,45^. In contrast to near-field scattering microscopy based on nanotips, our scheme is compatible with strong driving fields and we envision unprecedented insights to THz-driven nonlinear dynamics, such as interactions between polaritonic wavepackets^7,29^. Finally, we highlight the prospect of QFIM for imaging THz fields at the nanoscale using optical super-resolution microscopy^46^, paving a promising way towards ultrafast nanoscopy of strong electric fields inside nonlinearly driven nanosystems.

## Materials and methods

### Ultrafast QFIM microscope

We generate high-field single-cycle THz pulses by the tilted pulse front method^47^ in a MgO:LiNbO_3_ crystal using pulses from an amplified 10 kHz Yb-laser system (central wavelength 1030 nm, pulse energy 1 mJ), see Fig. S1 in the Supplementary Information. For the quantum dot excitation, we employ laser pulses from an optical parametric amplifier (OPA) at 530 nm or 480 nm wavelength, optimized for QFIM signal strength. The vertically polarized THz beam is focused on the sample with a 90°-off-axis parabolic gold mirror. We obtain a maximum field strength of 400 kV/cm in the sample plane and a peak frequency of ~0.9 THz via calibrated EO sampling using a 100 µm thick <110> GaP crystal. In addition, the THz field strength can be varied by polarization rotation of the pump pulses used for THz generation. The OPA beam provides wide-field excitation in the sample plane. Luminescence is collected by a microscope objective. We acquire luminescence images with a cooled CCD camera. The pump pulses used for THz generation are chopped at a few Hz, and we capture synchronized luminescence images with and without THz pumping. The consecutive image sequences are digitally subtracted to obtain the THz-induced difference signal. Ultrafast temporal resolution in this pump-probe scheme is obtained via scanning the temporal delay Δ*τ* between THz pump pulses and visible excitation pulses via a mechanical delay stage.

### Electromagnetic simulations

We employ a finite element solver (COMSOL Multiphysics) to calculate the electric near-fields of the structures. The model for the bowtie resonator consists of the gold antenna on a soda lime glass substrate^48,49^. For the propagating THz waveguide excitation, we employ a model consisting of two conducting metal bars (periodicity 50 µm, length 700 µm, gap 2 µm) on a soda lime glass substrate. We excite the structures using a plane wave single-cycle THz pulse (polarization perpendicular to the gap, center frequency 0.9 THz).

Details on the fabrication of gold microstructures, the synthesis of CdSe-CdS quantum dots and the polarization dependence of the bowtie antenna are presented in the Supplementary Information*.*

## Supplementary information

Supplementary Information

QFIM Movie of the THz bowtie antenna
