# Supplementary material for: Ultrafast imaging of terahertz electric waveforms using quantum dots
Source: Light Sci Appl. 2022 Jan 1;11:5. doi: 10.1038/s41377-021-00693-5 (PMC8720308; doi:10.1038/s41377-021-00693-5)
Supplement: Supplementary file 1 — Supplementary Information [file 41377_2021_693_MOESM1_ESM.docx]

**Supplementary Information for**

**Ultrafast Imaging of Terahertz Electric Waveforms using Quantum Dots**

Moritz B. Heindl^1^, Nicholas Kirkwood^2^, Tobias Lauster^3^, Julia A. Lang^1^,

Markus Retsch^3^, Paul Mulvaney^2^, and Georg Herink^1*^

^1^ Experimental Physics VIII – Ultrafast Dynamics, University of Bayreuth, Germany

^2^ ARC Centre of Excellence in Exciton Science, School of Chemistry, University of Melbourne, Australia

^3^ Physical Chemistry I, University of Bayreuth, Germany

**Contact data**

Moritz B. Heindl moritz.heindl@uni-bayreuth.de

Nicholas Kirkwood nrk@unimelb.edu.au

Tobias Lauster Tobias.Lauster@uni-bayreuth.de

Julia A. Lang Julia.A.Lang@uni-bayreuth.de

Markus Retsch markus.retsch@uni-bayreuth.de

Paul Mulvaney mulvaney@unimelb.edu.au

*Corresponding Author*

Georg Herink* georg.herink@uni-bayreuth.de

+49 921 / 55-3161

Universitaetsstr. 30, 95440 Bayreuth, Germany

**1. Figure S1 Details on the QFIM setup**

**
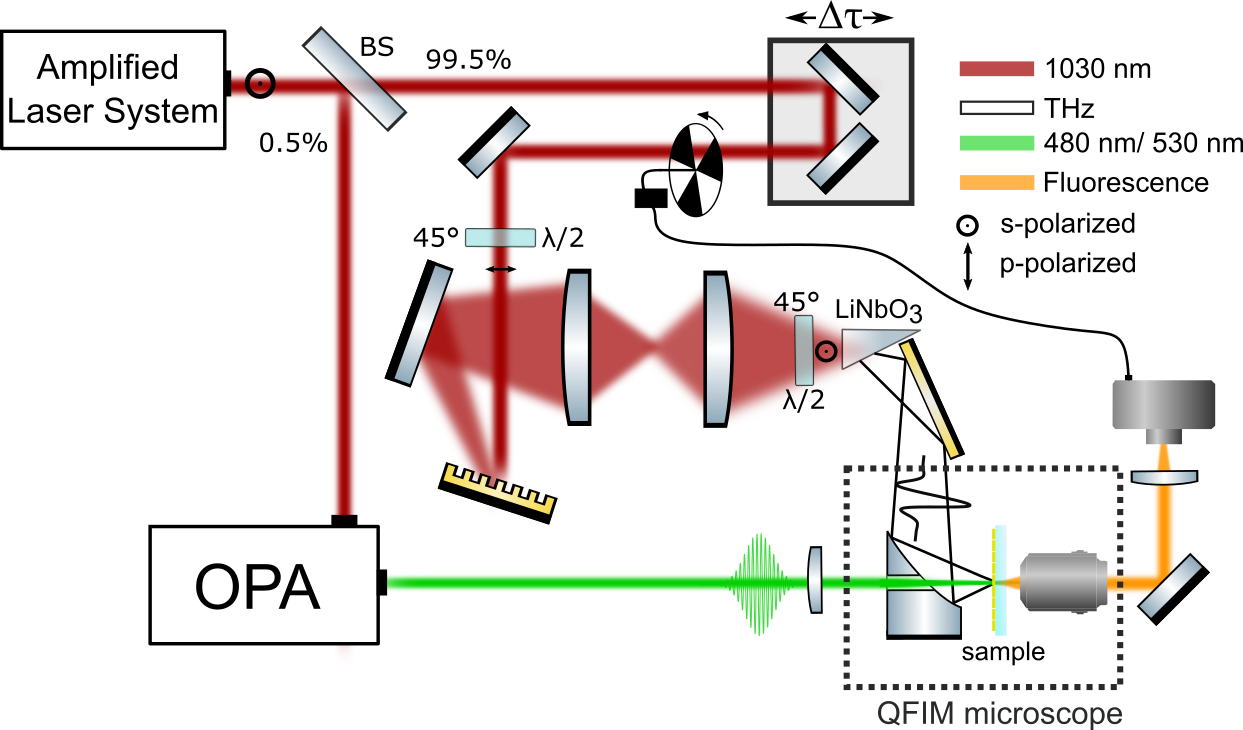
**

**Figure S1:** Experimental implementation of the QFIM setup.

**2. Extraction of the bowtie near-field waveform**

The waveform inside the bowtie antenna (Fig. 2 in the main article) can be extracted using an antenna model with a single resonance (1.2 THz) and the measured incident THz waveform as the driving field. The near-field waveform is converted into a modeled QFIM signal based on the nonlinear scaling (Fig. 3b in the main article) and the underlying waveform is matched to the experimental QFIM trace, obtaining the structural resonance behavior.


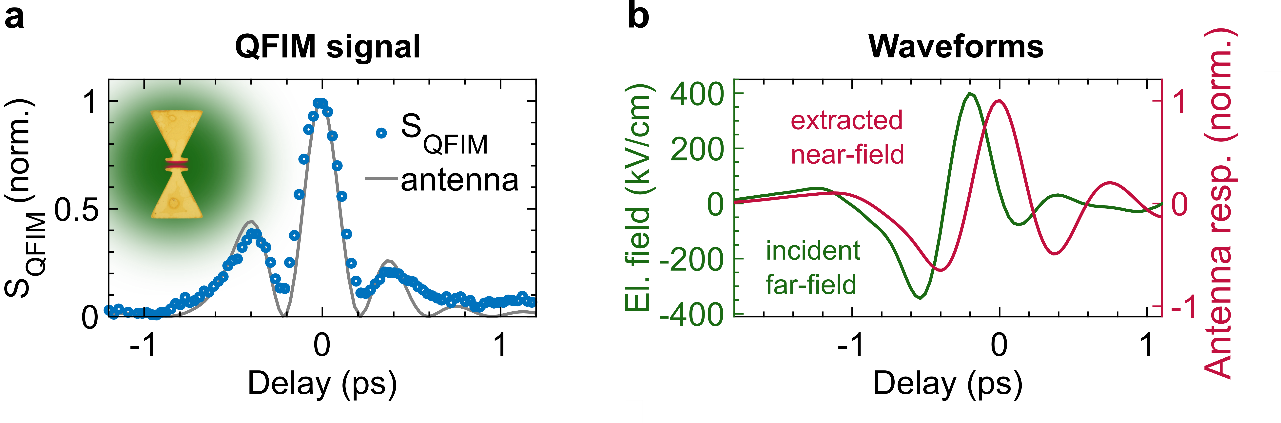


**Figure S2:** **a)** Local QFIM signal inside the gap of the bowtie antenna (blue circles) and the modeled temporal QFIM evolution (gray), derived from an antenna model. **b)** Comparison of the incident far-field and the extracted near-field.

**3. Spatial resolution of the microscope**

We estimate the spatial resolution of our setup using a line profile (Fig. S3a) from the cross-section of the QFIM image of the bowtie (Fig. 2c) as illustrated in Fig. S3. A Gaussian fit to the bright edge at the bowtie, resulting from strong field enhancement at the edge of the structure, yields a FWHM resolution of ~ 2 µm.


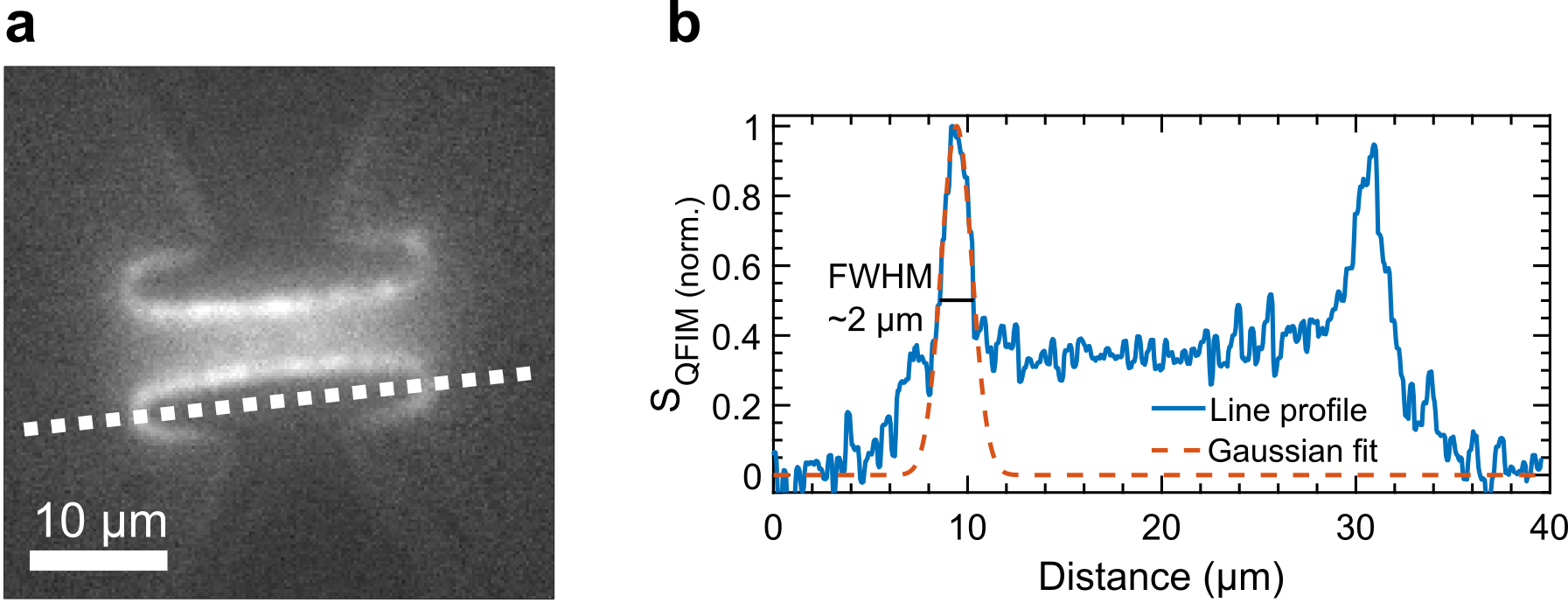


**Figure S3:** **a)** QFIM snapshot at maximum signal ($\Delta\tau=0 fs$), equivalent to Fig. 2c in the main article. The white dotted line indicates the cross-section used for the line profile in **b).**

**4. Bowtie near-fields and polarization dependence**

The local field enhancement of the bowtie antenna is sensitively dependent on the incident THz polarization. In the main article (Fig. 2), we discuss data obtained for polarization direction along the antenna axis. An additional measurement is presented in Fig. S4a with THz polarization nearly perpendicular to the antenna axis. We observe hot spots at the terminal elements and particularly at the extended edges of the antenna legs. These features are in close agreement with the electro-magnetic simulation, shown in Fig. S4b. We account for the slight rotation of the sample with respect to the incident THz polarization of ~6 °, creating a characteristic asymmetry in the signal pattern. The close correspondence to the QFIM data underlines the signal fidelity of the QFIM signal.


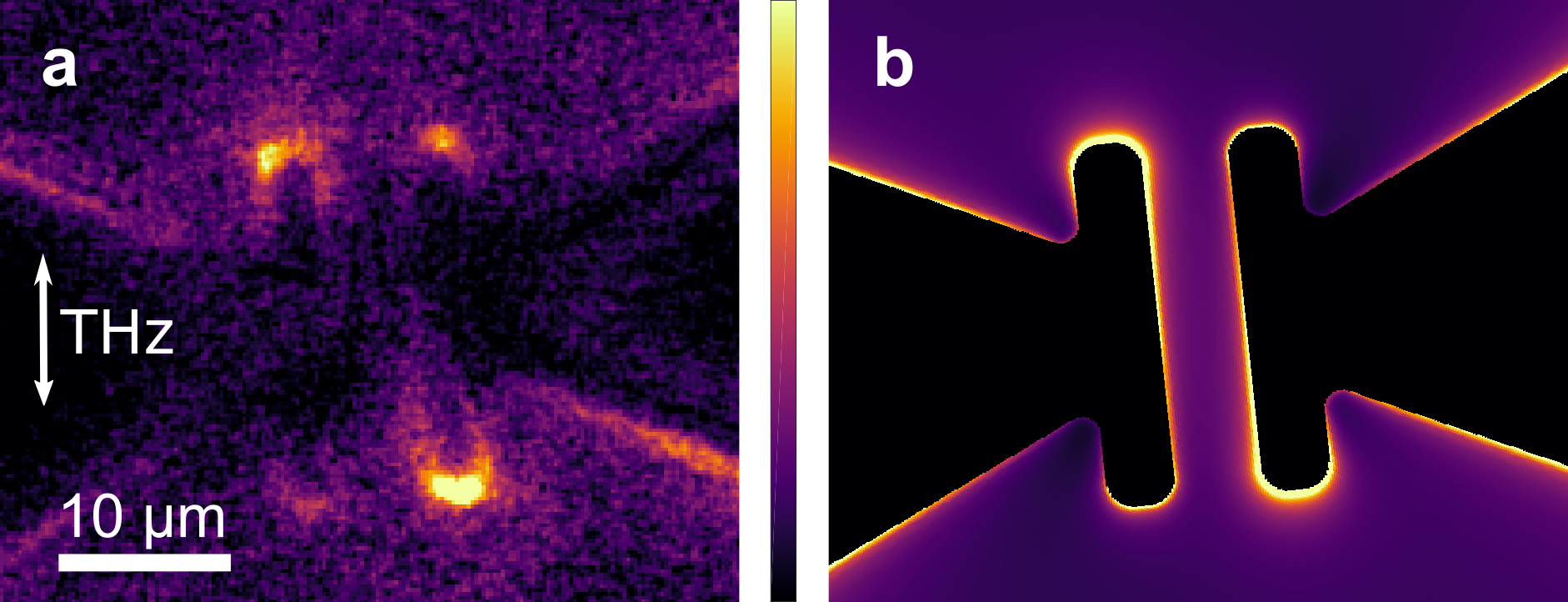


**Figure S4:** **a)** QFIM image at $\Delta\tau=0 fs$ (maximum signal) for incident THz polarization nearly parallel to the bowtie gap. **b)** Corresponding finite-element simulation of the electric field distribution.

**5. Details on quantum dots: synthesis and characterization**

**CdSe-CdS quantum dot synthesis**

We synthesize CdSe quantum dots following the method of Drijvers *et al.*^1^: A solution of cadmium phosphonate is prepared in a round bottom flask by heating a vacuum degassed solution of CdO (0.1925 g, 1.5 mmol), TDPA (1.67 g, 6 mmol), oleyl alcohol (6.44 g, 24 mmol), and TOPO (10 g) to 350 °C. Once the solution becomes colourless, 2 mL of TOP is injected. At a temperature returning to 350 °C, 1.7 mL of Se dissolved in TOP (1.48 mol L-1) is swiftly injected into the flask and the reaction is stopped by rapid cooling after the desired QD size is reached (4.3 nm diameter, first excitonic absorbance peak at 590 nm).

Next, we purify the CdSe quantum dots by precipitation with methanol and resuspension in toluene three times. Thick CdS shells are grown according to the method of Fan *et al*.^2^. Briefly, 176 nmol of CdSe QDs are suspended in a degassed solution of ODE (42 mL) and oleylamine (6 mL) and heated to 300 °C, followed by slow injection of cadmium oleate in ODE (0.22 mol L-1, 24 mL) and sulphur in TOP (0.23 mol L-1, 24 mL) over 4 hours. The solution is then heated to 310 °C and 5 mL of oleylamine is injected. Cadmium oleate in ODE (0.1 mol L-1, 24 mL) and octanethiol in ODE (0.1 mol L-1, 24 mL) are slowly injected over 2 hours. Subsequently, the solution is cooled and the QDs are purified by precipitation with acetone and resuspension in hexane three times.

**Chemicals**

Cadmium oxide (CdO, 99.95 %), trioctylphosphine oxide (TOPO, 90 %), trioctylphosphine (TOP, 97 %), octanethiol (97 %), selenium (pellets, ≥ 99 %) sulphur (≥ 99.98 %), oleylamine (technical grade 70 %), oleic acid (technical grade 90 %), oleyl alcohol (85 %) and octadecene (ODE, technical grade 90 %) is obtained from Sigma Aldrich and used without further purification. Tetradectylphosphonic acid (TDPA, ≥ 99 %) is obtained from PCI Synthesis and used without further purification.

**Quantum dot size distribution**

The quantum dot size distribution is characterized by analyzing the TEM image of a spin-coated sample at low quantum dot concentration as shown in Fig. S5.

The average diameter is ~11.0 nm (standard deviation 1.2 nm). Including the diameter of the quantum dot core (4.3 nm), the radius of the thick shell measures approximately 3.4 nm.


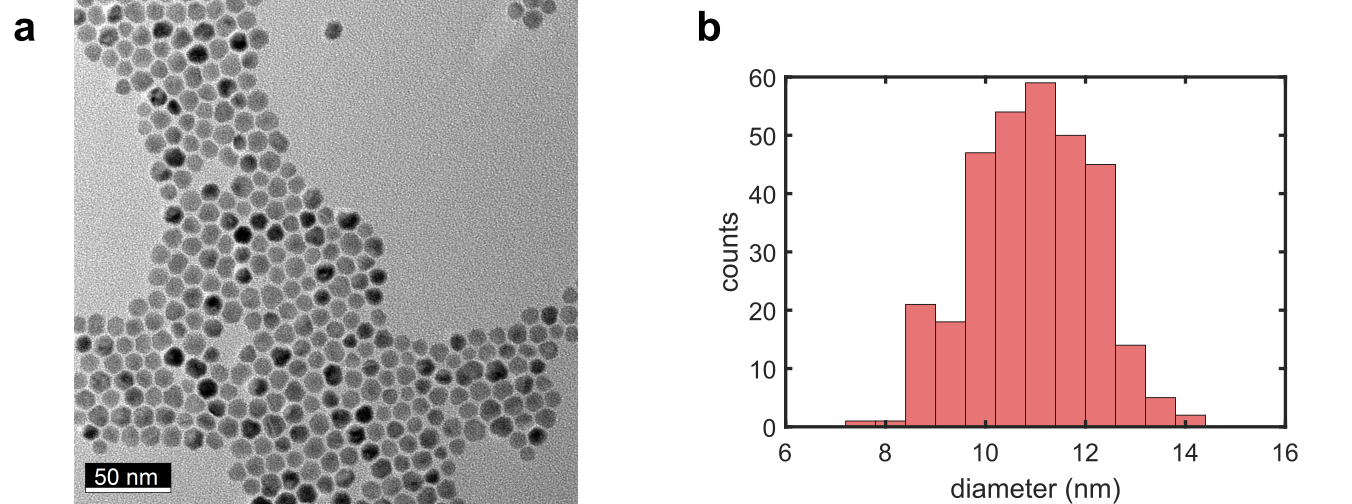


**Figure S5: (a)** Transmission electron micrograph of a quantum dot monolayer. (b) Histogram of the respective quantum dot size distribution.

**Absorption and emission spectra**

Visible spectra of quantum dot absorption and emission are displayed in Fig. S6. Additionally, the experimentally employed excitation wavelengths (bowtie: 530 nm, waveguide: 480 nm) and the employed longpass filter are indicated. The radiative quantum yield is ~60 % measured relative to Rhodamine 101.


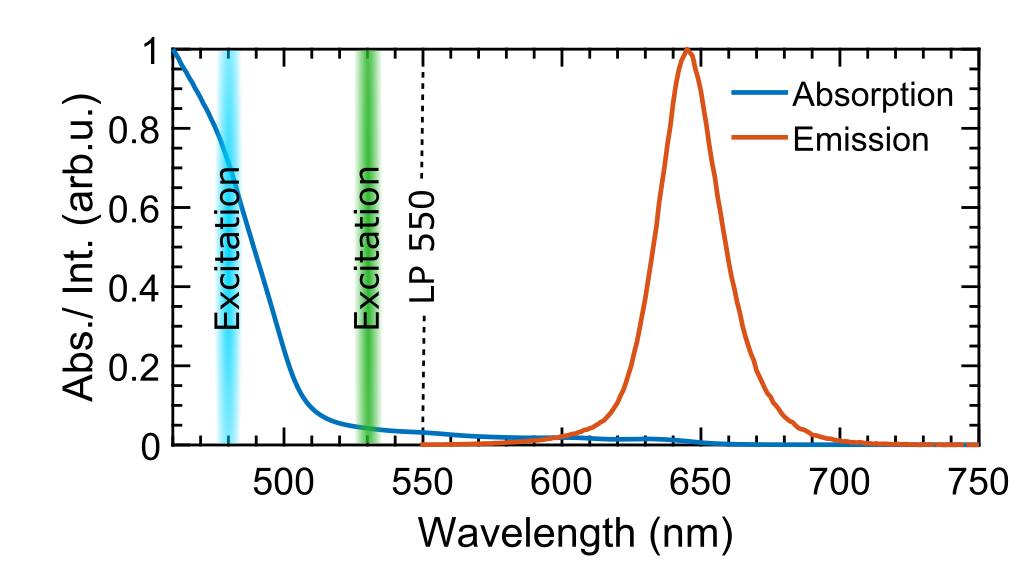


**Figure S6:** Absorption and emission spectra of the CdSe-CdS quantum dots.

**6. Fabrication of gold microstructures**

The gold microstructures are fabricated with a direct-write lithography instrument (Microwriter ML®3 Baby Plus, Durham Magneto Optics) in a double-layer lift-off process. The lift-off photoresist (LOR3B, Micro resist technology GmbH) is spin-coated at 3000 rpm for 30 s on a cleaned glass slide and a baking step on a hot plate is performed for 5 min at 90 °C. After cooling to room temperature a positive photoresist (Microposit® S1813, Micro resist technology GmbH) is spin-coated at 5000 rpm for 30 s. After a soft-baking step for 1 min at 100 °C, the sample is exposed to UV light (385 nm wavelength) with the direct-write lithography machine. The development is done with a Tetramethylammonium hydroxide based developer (Microposit® MIF-319, Micro resist technology GmbH) for 30 s. Immediately afterwards, the sample is submerged in ultrapure water, washed for 30 s and dried with compressed air.

Subsequently, approximately 2 nm chromium and 100 nm gold thin films are evaporated onto the samples using a physical vapor deposition system (BA 510 batch coating system, Balzers). During this process the metal fills the structure created with the lithography step. The removal of the photoresist and the additional metal yields the desired metal structure. The lift-off is done in 1-Methyl-2-Pyroolidone (VWR chemicals) by submersion and ultrasound treatment for 2 h.

**References**

1. Drijvers, E. *et al.* Revisited Wurtzite CdSe Synthesis: A Gateway for the Versatile Flash Synthesis of Multishell Quantum Dots and Rods. *Chem. Mater.* **28**, 7311–7323 (2016).

2. Fan, F. *et al.* Continuous-wave lasing in colloidal quantum dot solids enabled by facet-selective epitaxy. *Nature* **544**, 75–79 (2017).
